# A large-scale conformation sampling and evaluation server for protein tertiary structure prediction and its assessment in CASP11

**DOI:** 10.1186/s12859-015-0775-x

**Published:** 2015-10-23

**Authors:** Jilong Li, Renzhi Cao, Jianlin Cheng

**Affiliations:** Department of Computer Science, University of Missouri-Columbia, Columbia, MO 65211 USA; Informatics Institute, University of Missouri-Columbia, Columbia, MO 65211 USA; C. Bond Life Science Center, University of Missouri-Columbia, Columbia, MO 65211 USA

**Keywords:** Protein structure prediction, Sequence alignment, Template-based modeling, Template-free modeling, Model generation, Model evaluation

## Abstract

**Background:**

With more and more protein sequences produced in the genomic era, predicting protein structures from sequences becomes very important for elucidating the molecular details and functions of these proteins for biomedical research. Traditional template-based protein structure prediction methods tend to focus on identifying the best templates, generating the best alignments, and applying the best energy function to rank models, which often cannot achieve the best performance because of the difficulty of obtaining best templates, alignments, and models.

**Methods:**

We developed a large-scale conformation sampling and evaluation method and its servers to improve the reliability and robustness of protein structure prediction. In the first step, our method used a variety of alignment methods to sample relevant and complementary templates and to generate alternative and diverse target-template alignments, used a template and alignment combination protocol to combine alignments, and used template-based and template-free modeling methods to generate a pool of conformations for a target protein. In the second step, it used a large number of protein model quality assessment methods to evaluate and rank the models in the protein model pool, in conjunction with an exception handling strategy to deal with any additional failure in model ranking.

**Results:**

The method was implemented as two protein structure prediction servers: MULTICOM-CONSTRUCT and MULTICOM-CLUSTER that participated in the 11th Critical Assessment of Techniques for Protein Structure Prediction (CASP11) in 2014. The two servers were ranked among the best 10 server predictors.

**Conclusions:**

The good performance of our servers in CASP11 demonstrates the effectiveness and robustness of the large-scale conformation sampling and evaluation. The MULTICOM server is available at: http://sysbio.rnet.missouri.edu/multicom_cluster/.

**Electronic supplementary material:**

The online version of this article (doi:10.1186/s12859-015-0775-x) contains supplementary material, which is available to authorized users.

## Background

With the wide application of high-throughput next-generation sequencing technologies, the number of protein sequences is growing exponentially in the genomic era. Since protein functions are determined by protein structures, obtaining the structures of these proteins holds the key of utilizing this huge protein resource for biomedical research, bioengineering, and biotechnology development [1, 2].

Even though protein structures can be determined by experimental techniques such as x-ray crystallography and nuclear magnetic resonance (NMR), they can be only applied to solve the structures of a tiny portion of proteins due to their relatively high cost. Since the tertiary structure of a protein is almost uniquely specified by its amino acid sequence [3], computational methods of predicting protein structures from sequences are not only feasible, but also important to reduce the huge protein sequence-structure gap [4–8].

Computational protein structure prediction methods can be broadly classified into two categories: template-based modeling (TBM) [9–17] and template-free modeling (FM) [15, 18, 19]. Template-based modeling is based on the fact that evolutionarily related proteins tend to have similar structures [20] and structures change much slower than sequences [21]. Therefore, in order to predict the structure of a target protein, template-based modeling tries to find a target’s homologous protein with known structure and use it as a template, then transfer the structure of the template to the target based on their sequence alignment, and finally adjust the structure to account for the variation from the template sequence to the target sequence [22]. Thus far, template-based modeling is the most widely used and most accurate technique for protein structure prediction. However, it cannot work when no good template is found. In this situation, template-free modeling is needed to build protein structures from scratch or from the combination of small structural fragments. Even though template-based modeling and template-free modeling use very different techniques for protein structure prediction, they are in common in sampling protein conformations in a huge conformation space for a target. The former is just a more focused, targeted sampling based on known, related structural points in the space, whereas the latter is a more unbiased, random sampling to explore a large conformation space.

In order to improve the reliability and robustness of conformation sampling, some recent protein structure prediction methods start to enlarge the sampling space of template-based modeling rather than focusing on one or a few “best” points, and also try to integrate template-based modeling and template-free modeling when no good templates or only partial templates can be found for a target protein [23–26]. Based on our previous work of integrating multiple templates and alignments [23–25], we continued to develop and improve the large-scale conformation sampling approach to increase the diversity of template sampling, sequence alignment sampling, and model generation and to complement template-based modeling with template-free modeling in order to create a model pool of good quality. Given a pool of conformations for a target, another innovation is to apply an array of protein model quality assessment methods [27, 28] to evaluate the quality of the models and rank them rather than using only one or a few quality assessment methods as almost all other protein structure prediction methods do. Furthermore, we added a new exception handling protocol to detect the problems in the final model ranking in order to correct the errors slipped through the large-scale model evaluation.

We implemented the large-scale conformation sampling and evaluation approach as two automated MULTICOM web servers: MULTICOM-CONSTRUCT and MULTICOM- CLUSTER, which share the same conformation sampling protocol, but differ in the implementation of large-scale model quality assessment. We blindly benchmarked MULTICOM-CONSTRUCT and MULTICOM-CLUSTER in the 11th Critical Assessment of Techniques for Protein Structure Prediction (CASP11) in 2014. According to the CASP11 official assessment, the two servers were ranked among the best 10 server predictors for protein tertiary structure prediction and were effective for the targets of a wide-spectrum of difficulty.

## Methods

**Fig. 1:**
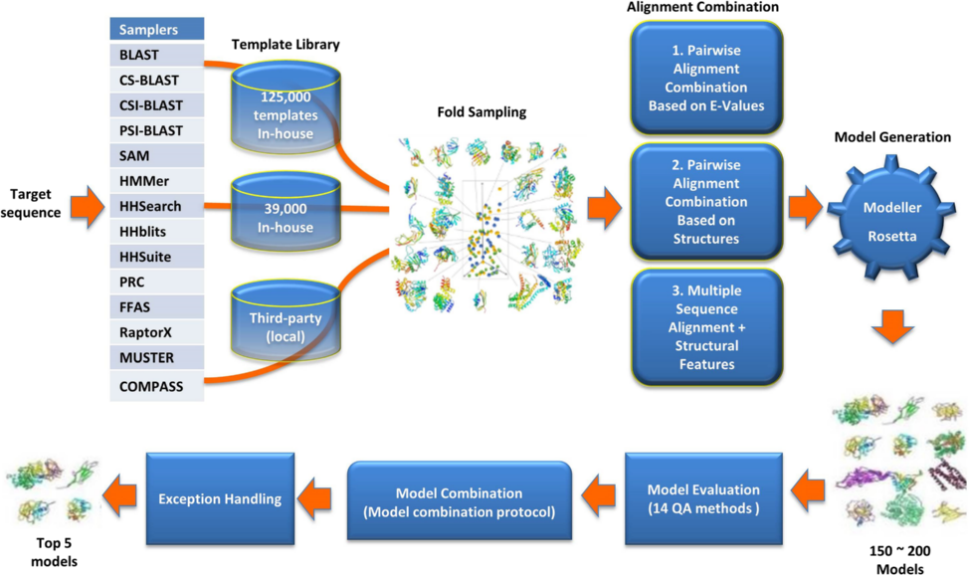
The large-scale model sampling and evaluation protocol of the MULTICOM protein structure prediction servers

Figure 1 illustrates the large-scale model sampling and evaluation method implemented in our servers (MULTICOM-CONSTRUCT and MULTICOM-CLUSTER). Given a target protein sequence, the method uses sequence-sequence alignment tools or sequence-profile alignment tools (e.g., PSI-BLAST [29], BLAST [29, 30], CS-BLAST [31], CSI-BLAST [31], SAM [32] and HMMer [33]) to search the sequence against a large template database consisting of ~125,000 proteins (a full copy of the PDB database [34] excluding the identical sequences), profile-profile alignment tools (e.g., HHSearch [35], HHSuite [35], HHblits [36], PRC [37], FFAS [38, 39] and COMPASS [40]) to search the sequence against a redundancy-reduced template database consisting of ~39,000 proteins, and locally installed MUSTER [41] and RaptorX [11] to search it against their smaller template databases. The parameters used with these alignment tools are described in Additional file 1: Table S1 in the supplemental document.

Each alignment tool identifies a list of templates and generates a list of pairwise target-template alignments. This template identification process corresponds to sampling templates for the target protein in the protein fold space approximated by the template protein databases.

The method uses three different ways to combine homologous templates and alternative alignments. *First*, it combines each pairwise target-template alignment (i.e. seed alignment) with other pairwise target-template alignments whose e-value is equal to or not much larger than that of the seed alignment. This central-star alignment algorithm of generating multiple sequence alignments from pairwise alignments is described in details in [42]. The multiple templates in this combination often have, but do not guaranteed to have the similar tertiary structures. *Second*, it combines each pairwise target-template alignment with other pairwise target-template alignments whose aligned structures are similar according to structural comparison. This approach combines one seed alignment with other pairwise alignments whose templates have similar structures with the template in the seed alignment using the central-star algorithm as in [24]. This approach guaranteed that the structures of the combined templates are consistent. *Third*, it generates a consensus list of templates ranked by the number of times they are selected by the alignment tools during template identification, and then uses several multiple sequence alignment tools (e.g., MUSCLE [43, 44], MSACompro [45], and MSAProbs [46]) to align the target with the top consensus template proteins in order to generate multiple sequence alignments.

The combined target-template alignments together with template structures are fed into Modeller [47, 48] to generate structural models using comparative modeling. For the targets without reliable templates identified, a template-free modeling tool Rosetta [26, 49] is called to generate dozens of models to complement the template-based models. Targets that contain both easy (template-based) and hard (template-free) domains are often decomposed into different chunks by dividing the target sequence into several sub-sequences (chunks) according to the sequence alignments, where easy domains are covered by homologous templates (i.e., e-value < 1) and hard domains aren’t covered by any homologous template. Different modeling protocols (i.e., template-based protocol or template-free protocol) are chosen to predict the structures of each chunk. The conformations of all the chunks will be combined into a full-length model using Modeller [47, 48] by using the structural models of different chunks as the templates for the target protein. In total, about 150–200 structural models are generated for a target using the protocol described above.

The pool of predicted models was evaluated by 14 quality assessment (QA) methods (e.g., MULTICOM-NOVEL_QA – a new in-house single QA method, ModFOLDclust2 [50], ProQ2 [51], Pcons [52], APOLLO [53], ModelEvaluator [54], ModelCheck2 – an improved version of ModelEvaluator, QApro – a weighted combination of ModelEvaluator and APOLLO, SELECTpro [55], Dope [56], DFIRE2 [57], OPUS_PSP [58], RWplus [59], RF_CB_SRS_OD [60]). MULTICOM-CONSTRUCT used the consensus (average) ranking of the individual rankings produced by these methods to select top five models as predictions [27, 28]. MULTICOM-CLUSTER selected top five models based on primarily Apollo pairwise similarity score in conjunction with the coverage and identify of template-target alignments, the e-values of alignments used to generate the models, and the types (i.e., template-based, template-free, or the combination of the two) of the models.

No matter how comprehensive the model evaluation process is, some bad models may still be ranked at the top occasionally. In order to solve the problem, for the first time, we designed an exception handling strategy to improve or replace the bad models within top five models in the following six situations: (**1**) If the top one model is a template-based model and > = 40 residues in its front end or back end are not covered by (i.e. aligned with) any template, the conformation of these uncovered residues will be replaced by the conformation of another model that is covered by a template. (**2**) If the top one model is a template-based model and < 40 % of residues are covered by a template, the model will be replaced by another top-ranked model with > = 40 % template coverage. (**3**) If the top one model is template-based and the coverage of the most significant template is < 30 %, all other templates’ coverage is < 50 % and the highest average pairwise similarity score in the model pool is < 0.2 (i.e. a hard modeling case), the model is replaced by another top-ranked model if available. (**4**) If a model with > 0.7 target-template sequence identity and > 0.8 template coverage exists in the model pool and the highest average pairwise model similarity is > 0.4 GDT-TS score (i.e. an easy modeling case), the top one model is replaced with the model that has highest target-template sequence identity and > 0.8 template coverage and the highest average pairwise GDT-TS score. (**5**) If the top ranked model is a combination of models of protein domains and a significant template with > 0.7 coverage is found, the top model may be replaced by a highly ranked model without domain combination. If domain division and combination happens, we check if the top domain-based model is better than the full-length model to decide if domain division and combination has to be reverted. If the e-value of templates in top ranked full-length models (e.g. HHSearch and RaptorX models) is < e-6, the coverage of templates is > 0.7, and the top GDT-TS score between the models is > 0.35, the top full-length model will replace the top domain-based model as new top 1 model, and the domain-based model will be used as no. 5 model. And (**6**) If all the top five MULTICOM-CONSTRUCT models are *ab initio* models, no. 4 and no. 5 models are replaced with top two template-based models in order to increase the diversity of the submitted models.

## Results and discussion

### Summary of results

The method was implemented as two protein structure prediction servers: MULTICOM-CONSTRUCT and MULTICOM-CLUSTER. MULTICOM-CONSTRUCT and MULTICOM-CLUSTER participated in the 11th Critical Assessment of Techniques for Protein Structure Prediction (CASP11) in 2014. According to the CASP11 official assessment at http://www.predictioncenter.org/casp11/zscores_final.cgi (click on server groups), MULTICOM-CONSTRUCT and MULTICOM-CLUSTER were ranked among best 10 methods (no. 6 and no. 7) for protein tertiary structure prediction among 44 server predictors.

We evaluated MULTICOM-CONSTRUCT and MULTICOM-CLUSTER on the 105 CASP11 domains whose experimental structures were released to date. The difficulty of these domains ranges from easy template-based modeling to hard template-free modeling. Our submitted server models for 105 CASP11 domains were superimposed onto the true structures. GDT-TS scores and TM-scores of the models were calculated by the TM-score program [61]. Table 1 reports the average GDT-TS scores and TM-scores of top one and best of five models predicted by our servers. The average TM-Scores of the first submitted models and the best of five models are 0.54 and 0.56 respectively for the two servers, which are higher than the commonly accepted threshold of 0.5 for a correct topology. Table 2 reports the number of target domains for which our servers submitted models to CASP11 whose TM-Scores are higher than 0.5, a common threshold indicating if a model has correct topology. Our server submitted models with a TM-Score higher than 0.5 for ~75 % of the TBM domains. But TM-Scores of almost all the models submitted for FM domains are lower than 0.5, suggesting that generating or selecting good models for FM targets is still a major challenge.

**Table 1:** Average GDT-TS scores and TM-scores of MULTICOM-CONSTRUCT and MULTICOM-CLUSTER models on 105 CASP11 domains

Predictors	Top one		Best of top five	
	GDT-TS	TM-score	GDT-TS	TM-score
MULTICOM-CONSTRUCT	0.48	0.54	0.50	0.56
MULTICOM-CLUSTER	0.49	0.54	0.50	0.56

The numbers represent the average GDT-TS scores and TM-scores of top one and best of top five models predicted by MULTICOM-CONSTRUCT and MULTICOM-CLUSTER on 105 CASP11 domains

**Table 2:** The number of target domains whose models have TM-Scores higher than 0.5

Predictors	TBM domains (75)		FM domains (30)	
	Top 1	Best of 5	Top 1	Best of 5
MULTICOM-CONSTRUCT	57	59	0	0
MULTICOM-CLUSTER	54	57	0	1

The numbers represent the number of target domains for which MULTICOM-CONSTRUCT and MULTICOM-CLUSTER submitted models to CASP11 whose TM-Scores with native structures are higher than 0.5 on 75 CASP11 TBM domains and 30 FM domains

### The quality of the model pool

**Fig. 2:**
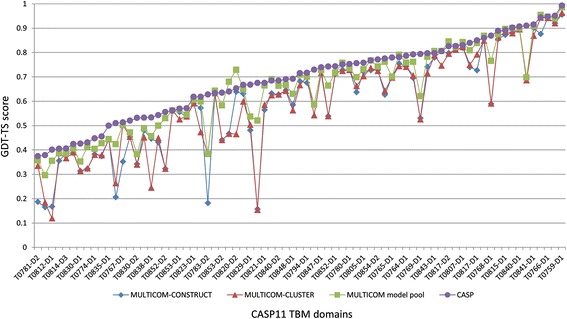
Comparison of top 1 models in the MULTICOM servers and CASP11 on 75 TBM domains. The comparison is based on GDT-TS scores of top 1 models of MULTICOM-CONSTRUCT, top 1 models of MULTICOM-CLUSTER, the best models in the MULTICOM model pool, and best of top 1 models in CASP11 on 75 easy TBM CASP11 domains

**Fig. 3:**
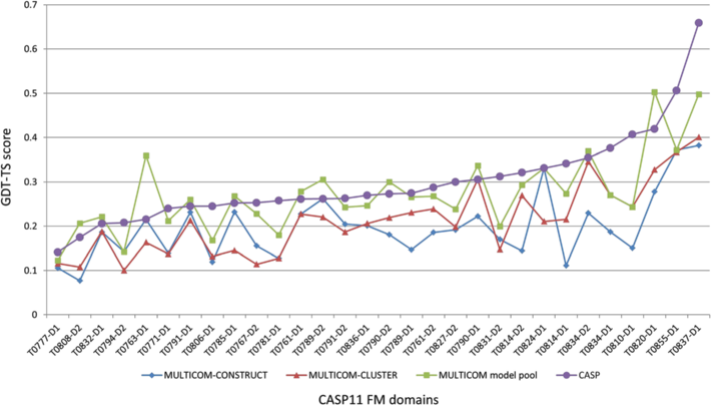
Comparison of top 1 models in the MULTICOM servers and CASP11 on 30 FM domains. The comparison is based on GDT-TS scores of top 1 models of MULTICOM-CONSTRUCT, top 1 models of MULTICOM-CLUSTER, the best models in the MULTICOM model pool, and best of top 1 models in CASP11 on 30 hard FM CASP11 domains

**Fig. 4:**
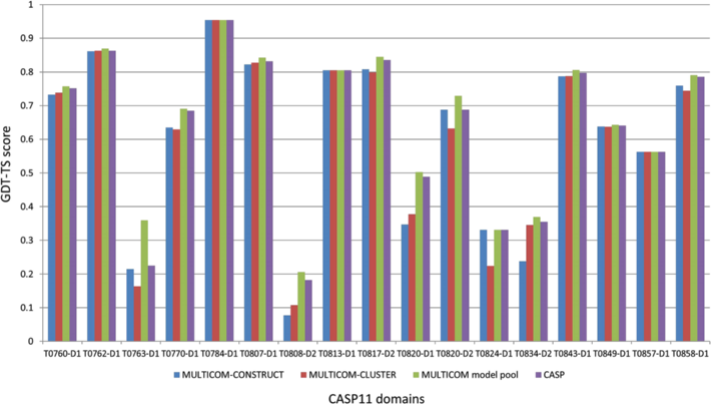
Comparison of top 5 models in the MULTICOM servers and CASP11 on 17 domains. The comparison is based on GDT-TS scores of best of top 5 models of MULTICOM-CONSTRUCT, best of top 5 models of MULTICOM-CLUSTER, the best models in the MULTICOM model pool, and best of top 5 models in CASP11 on 17 CASP11 domains where the best models in our model pool had the same (higher) GDT-TS scores as (than) best of top 5 models in CASP11

We investigated the quality of the pool of conformations for each target generated by our servers in comparison with our submitted models and all the CASP11 models submitted by up to 44 server groups around the world. Figures 2 and 3 show the comparison of GDT-TS scores of top 1 models of MULTICOM-CONSTRUCT, top 1 models of MULTICOM-CLUSTER, the best models in the MULTICOM model pool, and the best of top 1 models in CASP11 on 75 easy TBM domains and 30 hard FM domains separately. The target domains were sorted by the scores of the best CASP11 models, which are some sort of indicators of the difficult of the target domains. From the figures, the best models in our model pool had the same (higher) GDT-TS scores as (than) the best of top 1 models in CASP11 on 16 TBM domains and 12 FM domains respectively. Figure 4 shows the comparison of GDT-TS scores of best of top 5 models of MULTICOM-CONSTRUCT, best of top 5 models of MULTICOM-CLUSTER, the best models in the MULTICOM model pool, and best of top 5 models in CASP11 on 17 CASP11 domains (12 easy TBM domains and 5 hard FM domains) where the best models in our model pool had the same (higher) GDT-TS scores as (than) best of top 5 models in CASP11. Also, the differences in GDT-TS scores between the best models in our model pool and the best models in the CASP11 model pool produced by dozens of protein structure prediction methods in the community are less than 0.02 and 0.05 on 40 and 65 domains separately. The results indicate that our large-scale conformation sampling method can generate good models for a large portion of targets.

Moreover, we evaluated a number of alignment tools used by the MULTICOM servers. Table 3 reports how many times each of the independent pairwise alignment tools excluding the alignment combination methods generated alignments leading to the creation of the best models for 75 TBM and 30 FM CASP11 domains. From the table, HHSearch / HHSuite and its variants contributed to the creation of the best models for 41 TBM and 8 FM domains, and performed best among these methods on both TBM and FM domains. However, it is worth noting that the MULTICOM servers used several different versions of HHSearch and their combined results were reported here. RaptorX, MUSTER, HHblits and COMPASS also contributed to the generation of the best models on both TBM and FM domains. In addition to these alignment tools, the template-free modeling tool Rosetta generated the best models for 1 TBM domain and 13 FM domains. The alignment and model combination algorithms in the MULTICOM server that combined the output of the independent alignment tools also generated best models for 24 TBM domains and 4 FM domains. The experiment shows that the combination of multiple different alignment tools improves the quality of the best models in the model pool and is an effective way to improve the reliability and robustness of protein structure prediction.

**Table 3:** The number of times that each alignment tool contributed to generation of the best models

Alignment tool	# of times generating best models		
	TBM & FM domains	TBM domains	FM domains
HHSearch / HHSuite	49	41	8
RaptorX	13	10	3
MUSTER	7	5	2
HHblits	7	6	1
COMPASS	6	5	1
PSI-BLAST	2	2	
BLAST	1	1	
HMMer	1	1	
PRC	1	1	
FFAS	1	1	

The numbers represent the number of times that each of the independent pairwise alignment tools excluding the alignment combination methods generated alignments leading to the creation of the best models for 75 TBM and 30 FM CASP11 domains. It is worth noting that the results of several versions of HHSearch used in the MULTICOM server are combined together

### Evaluation of the large-scale model ranking strategy

**Fig. 5:**
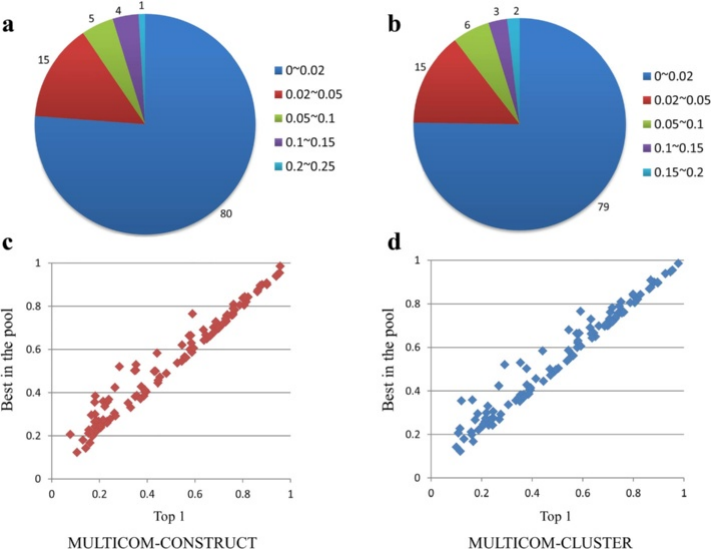
Evaluation of the ranking strategy. **a** and **b** illustrate the number of domains in various ranges of differences in GDT-TS scores between best of top 5 models and top 1 models generated by MULTICOM-CONSTRUCT and MULTICOM-CLUSTER respectively on 105 CASP11 domains. **c** and **d** show scatter plots of GDT-TS scores between top 1 models and the best models in the model pool for the two servers separately

We compared top 1 models with best of top five models and the overall best models in the model pool for MULTICOM-CONSTRUCT and MULTICOM-CLUSTER on 105 CASP11 domains in order to check the performance of the ranking strategy. Figure 5 (a) and (b) illustrates the number of domains in various ranges of differences of GDT-TS scores between best of top 5 models and top 1 models generated by the two servers respectively. The differences of GDT-TS scores are small (i.e. < 0.02) on 80 and 79 domains, and the top 1 models are the best of top five models on 43 and 41 domains for MULTICOM-CONSTRUCT and MULTICOM-CLUSTER separately. So, the ranking strategy worked generally well. However, it failed on some domains. For example, the top 1 model of T0816-D1 selected by MULTICOM-CONSTRUCT had a GDT-TS score 0.47, 0.21 less than 0.68 of the best of top five models. Figure 5 (c) and (d) shows scatter plots of GDT-TS scores between top 1 models and the best models in the model pool. The differences of GDT-TS scores are less than 0.02 on 46 and 50 domains, and less than 0.05 on 74 and 79 domains for MULTICOM-CONSTRUCT and MULTICOM-CLUSTER separately. Therefore, the ranking strategy successfully picked up good models on most of the domains.

We also evaluated the performance of 14 model quality assessment methods and their consensus ranking. The consensus ranking of a model is the average rank of all the rankings predicted by these methods for the model. Table 4 reports the number of times when top 1 models selected by an individual quality assessment (QA) method were actually the best of top 1 models identified by all the QA methods, and the number of times when top 1 models selected by an individual method were actually the best models in the MULTICOM model pool. “Avg loss” means the average loss (difference) in GDT-TS scores between the best models and top 1 models ranked by each QA method. A tolerance of marginal difference in scores is applied when calculating these numbers. The past CASP QA assessments [62–65] show that few predictors could consistently identify good models within 0.02 GDT-TS score from the best models, and the average loss of GDT-TS score of best QA methods was generally larger than 0.02. Therefore, in our experiment, if GDT-TS score of the top 1 model identifies by an individual method was within 0.02 GDT-TS score from the actual best model, the method is considered to successfully identify the best model. Also, we removed targets whose highest GDT-TS score is < 0.35 for the analysis because these models are of poor quality and GDT-TS score is not a good measure for differentiating models of less than 0.35 GDT-TS score. The table shows that the consensus ranking performed better than 14 individual QA methods in terms of selecting the top 1 model. SELECTpro, ModFOLDclust2, APOLLO, Pcons, and ProQ2 performed best among the 14 individual methods. However, the probability of any of these methods selecting the best model is low, indicating that selecting the best model is still more or less a guess game.

**Table 4:** Comparison of 14 model quality assessment methods and their consensus ranking

QA method	Best of top 1	Avg loss	Best in the pool	Avg loss
Consensus ranking	34	0.04	17	0.07
SELECTpro	32	0.05	17	0.08
ModFOLDclust2	30	0.07	18	0.10
APOLLO	30	0.07	16	0.09
Pcons	29	0.07	16	0.10
ProQ2	27	0.05	15	0.07
QApro	18	0.07	8	0.09
ModelCheck2	16	0.16	10	0.18
MULTICOM-NOVEL_QA	11	0.11	4	0.14
DFIRE2	9	0.11	6	0.14
Dope	9	0.11	6	0.14
ModelEvaluator	9	0.13	6	0.16
OPUS_PSP	9	0.11	6	0.14
RF_CB_SRS_OD	9	0.11	6	0.14
RWplus	9	0.11	6	0.14

“Best of top 1” means the number of times when top 1 models selected by an individual QA method were actually the best of the top 1 models identified by all the QA methods. “Best in the pool” means the number of times when top 1 models by an individual method were actually the best models in the MULTICOM model pool. “Avg loss” means the average loss of GDT-TS scores between the best models and top 1 models ranked by each QA method

### Case study

From our analysis, the submitted models of MULTICOM-CONSTRUCT and MULITICOM-CLUSTER are the best models among all the CASP11 server models on six CASP11 domains: T0762-D1 (TBM), T0784-D1 (TBM), T0813-D1 (TBM), T0820-D2 (TBM), T0824-D1 (FM), and T0857-D1 (TBM).

**Fig. 6:**
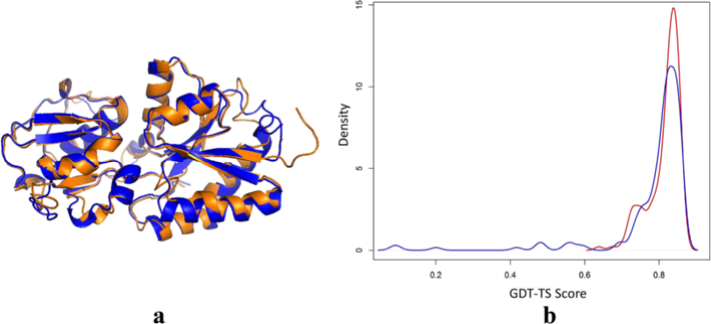
One good prediction of MULTICOM-CLUSTER on T0762-D1. **a** Structural superposition between the native structure of T0762-D1 (*blue*) and the no. 3 model of MULTICOM-CLUSTER (*gold*). **b** Distribution of GDT-TS scores of the MULTICOM server models (*red*) and the CASP server models (*blue*) of T0762-D1

Figure 6 (a) shows the structural superposition between the native structure of T0762-D1 (blue) and a high-accuracy model (no. 3) predicted by MULTICOM-CLUSTER (gold), which was reconstructed from multiple templates (i.e. 4IB2A, 4EF1A, 4OTEA, and 4K3FA). The model is the best model among all the models submitted to CASP11 for T0762-D1. It has a GDT-TS score 0.86 and RMSD 2.3 Å with the native structure. Figure 6 (b) illustrates the distributions of GDT-TS scores of the MULTICOM server models (red) and the CASP server models (blue) of T0762-D1. Here, the MULTICOM server models include all the models in the MULTICOM candidate pool. Density (Y-axis) represents the number of models. The two distributions are similar and most models have GDT-TS scores around 0.8 or above, but the MULTICOM model pool contains the best models. The results show that our method successfully identified homologous templates, generated good alignments, and constructed and picked up high-quality models for this domain.

**Fig. 7:**
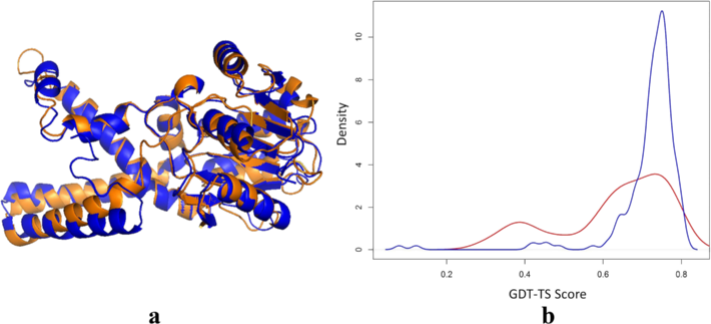
One good prediction of MULTICOM-CONSTRUCT on T0813-D1. **a** Structural superposition between the native structure of T0813-D1 (*blue*) and the top 1 model of MULTICOM-CONSTRUCT (*gold*). **b** Distribution of GDT-TS scores of the MULTICOM server models (*red*) and the CASP server models (*blue*) of T0813-D1

Figure 7 (a) shows the structural superposition between the native structure of T0813-D1 (blue) and the top 1 model of MULTICOM-CONSTRUCT (gold), which was reconstructed from four templates (3KTDA, 2F1KA, 3B1FA, and 3GGOA). The model is the best model among all the models submitted to CASP for T0813-D1. It has a GDT-TS score of 0.81 with the native structure. Figure 7 (b) illustrates the distributions of GDT-TS scores of the MULTICOM server models (red) and the CASP server models (blue). Here, the MULTICOM server models include all the models in the MULTICOM candidate pool. Density (Y-axis) represents the number of models. The distribution of the MULTICOM server models is bimodal, suggesting the models were constructed from both very good templates and some sub-optimal templates. The distribution of the CASP server models is uni-modal with mostly good models and a small number of low-quality models that may be constructed by template-free modeling methods or from bad templates. The MULTICOM server model is the best server model for T0813-D1 in CASP11, indicating that our method generated a pool of good models and selected the best models from the pool for this domain.

**Fig. 8:**
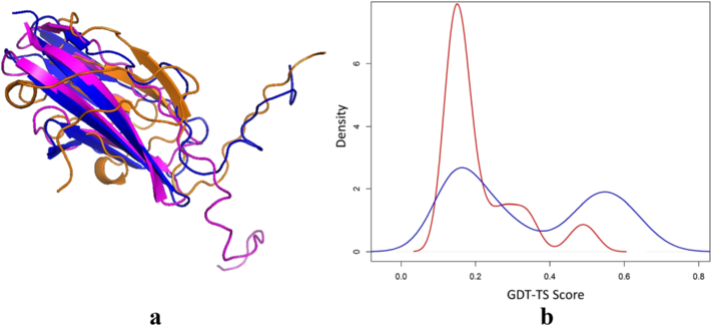
One bad prediction of MULTICOM-CLUSTER on T0845-D1. **a** Structural superposition between the native structure of T0845-D1 (*blue*) and the no. 2 submitted model of MULTICOM-CLUSTER (*gold*) and the best model in the MULTICOM model pool (*purple*). **b** Distribution of GDT-TS scores of the MULTICOM server models (*red*) and the CASP server models (*blue*) of T0845-D1

We also investigated the cases in which the MULTICOM servers failed due to not generating good models or not being able to select good models from the model pool. The most dramatic failure occurred on T0845-D1, for which the best submitted model (no. 2) by MULTICOM-CLUSTER has a GDT-TS score of 0.29. The GDT-TS score of the best model in the MULTICOM model pool is 0.52, 0.23 point higher than the best submitted model. Figure 8 (a) shows the structural superposition between the native structure of T0845-D1 (blue) and the best submitted model by MULTICOM-CLUSTER (gold) and the best model in the MULTICOM model pool (purple). The best model in the pool superimposed much better with the native structure. Figure 8 (b) visualizes the distributions of GDT-TS scores of the MULTICOM server models (red) and the CASP server models (blue) of T0845-D1. The majority of the MULTICOM server models except a few ones are of bad quality. The distribution of the CASP server models is bimodal, where a significant portion of models is of good quality. The GDT-TS score 0.71 of the best CASP11 model (TASSER-VMT_TS4) is much better than that of MULTICOM models, suggesting that our servers failed to generate good models. Also, the ranking strategy in our servers was not able to select the few relatively good models in its model pool on this domain. The case suggests that both model generation and model selection in the MULTICOM servers still have a significant room for improvement.

### Availability

After being rigorously tested in CASP11, the protein structure prediction web service of MULTICOM-CLUSTER is released for public use at http://sysbio.rnet.missouri.edu/multicom_cluster/. Since MULTICOM-CONSTRUCT is slower than MULTICOM-CLUSTER and has similar accuracy as MULTICOM-CLUSTER, it is not made available for public use.

The experimental data of MULTICOM in CASP11 is available through the link “Experimental data (models and alignments) in CASP11” on the home page of the MULTICOM server at http://sysbio.rnet.missouri.edu/multicom_cluster/.

## Conclusions

We developed and implemented a large-scale conformation sampling and evaluation method to improve the reliability and robustness of protein structure prediction, overcoming the problem of failing to obtain the best template, alignment and model in traditional protein structure prediction methods. The approach can naturally integrate multiple templates, multiple alignments, and diverse sampling and evaluation methods into one system to improve model sampling and ranking as demonstrated by the good performance of our method in the CASP11 experiment. Furthermore, our analysis of the quality of conformation pool provides the new insights into the sampling and evaluation of protein models. Overall, the method and its server implementation are useful tools for protein structure predictors and users.

However, despite of the progress enabled by the large-scale sampling and evaluation approach, there are still some major challenges in protein structure prediction, including how to reliably identify weak homologous templates from irrelevant noisy templates, how to enrich the proportion of good alignments, how to distinguish a few good models from a large number of low-quality models, and finally how to generate better template-free models when no homologous template exists. In order to solve these problems, on the one hand more sensitive or complementary data mining methods need to be developed to mine a large number of templates, alignments, and protein models produced by existing methods, on the other hand novel methods for simulating alignments and structural models for hard targets more effectively are required to generate ensembles of protein conformations of better quality.

## Additional file

Additional file 1: Table S1. It contains the information about different sequence alignment tools used in our method. The information includes names, version numbers, alignment types (e.g. sequence-sequence, profile-sequence, and profile-profile), and parameters of these sequence alignment tools. (DOCX 19 kb)
