# Supplementary material for: A large-scale conformation sampling and evaluation server for protein tertiary structure prediction and its assessment in CASP11
Source: BMC Bioinformatics. 2015 Oct 23;16:337. doi: 10.1186/s12859-015-0775-x (PMC4619059; doi:10.1186/s12859-015-0775-x)
Supplement: Additional file 1: Table S1. — It contains the information about different sequence alignment tools used in our method. The information includes names, version numbers, alignment types (e.g. sequence-sequence, profile-sequence, and profile-profile), and parameters of these sequence alignment tools. (DOCX 19 kb) [file 12859_2015_775_MOESM1_ESM.docx]

The supplemental document for the paper “A Large-Scale Conformation Sampling and Evaluation Server for Protein Tertiary Structure Prediction and its Assessment in CASP11”

Jilong Li, Renzhi Cao, Jianlin Cheng

Computer Science Department, University of Missouri, Columbia, MO 65211, USA

Table S1. The information about different sequence alignment tools used in our method. The information includes names, version numbers, alignment types (e.g. sequence-sequence, profile-sequence, and profile-profile), and parameters of these sequence alignment tools.

| Alignment tool | Version number | Type | Parameters |
| --- | --- | --- | --- |
| CSBLAST | 2.1.0 | Sequence-sequence | -j 5 –e 1 –h 0.0000000001 |
| CSIBLAST | 2.1.0 | Sequence-sequence | -j 5 –e 1 –h 0.001 |
| BLAST | 2.2.17 | Sequence-sequence | -F T -j 5 -e 1 |
| PSI-BLAST | 2.2.17 | Profile-sequence | -j 5 –e 1 –h 0.001 |
| SAM | 3.5.i686 | Profile-sequence | -calibrate 1 –sw 2 –dpstyle 0 –adpstyle 5 |
| HMMer | 3.0b3 | Profile-sequence | Default parameters |
| FFAS | 03 | Profile-profile | Default parameters |
| COMPASS | 2.42 | Profile-profile | -e 1 |
| PRC | 1.5.6 | Profile-profile | -hits 50 –align prc |
| MUSTER | 3.0 | Profile-profile | Default parameters |
| RAPTOR | 1.66 | Profile-profile | Number of models is set to 10 |
| HHSearch | 1.2 | Profile-profile | Default parameters |
| HHSearch | 1.5.0 | Profile-profile | Default parameters |
| HHSearch | 1.5.1 | Profile-profile | -realign –mact 0 |
| HHSearch in HHSuite | 2.0.8 | Profile-profile | -realign –mact 0 |
| HHblits | 2.2.17 | Profile-profile | -oa3m –n $nr_iteration and –realign –mact 0 |
